# Trends and patterns of antibiotic consumption in China’s tertiary hospitals: Based on a 5 year surveillance with sales records, 2011-2015

**DOI:** 10.1371/journal.pone.0190314

**Published:** 2017-12-27

**Authors:** Haishaerjiang Wushouer, Ye Tian, Xiao-Dong Guan, Sheng Han, Lu-Wen Shi

**Affiliations:** 1 Department of Pharmacy Administration and Clinical Pharmacy, School of Pharmaceutical Science, Peking University, Beijing, China; 2 International Research Center for Medicinal Administration (IRCMA), Peking University, Beijing, China; Purdue University, UNITED STATES

## Abstract

The consumption of antibiotics is a major driver in the development of antimicrobial resistance. This study aims to identify the trends and patterns of the total antibiotic consumption in China’s tertiary hospitals from 2011 to 2015 by retrospectively analyzing aggregated monthly surveillance data on antibiotic sales made to 468 hospitals from 28 provinces. Antibiotic consumption was expressed in DDD per 1,000 inhabitants per day (DID). We compared population weighted antibiotic consumption patterns in China with European countries using indicators from the European Surveillance of Antimicrobial Consumption (ESAC). Total antibiotic consumption, including all the specific antibiotic class except for aminoglycoside antibacterials, were significantly increased during the study period from an average of 7.97 DID in 2011 to 10.08 DID in 2015. In 2015, the eastern regions of China consumed the most antibiotics using population denominator while the western regions consumed the most using inpatient denominator. Cephalosporins accounted for 28.6% of total DID, followed by beta-lactam-beta-lactamase inhibitor combinations (20.0%), macrolides (17.4%), and fluoroquinolones (10.5%). Antibiotic in parenteral form accounted for nearly half of all antibiotics. Although over the past few years major efforts had been made to reduce the risks of excessive antibiotic use through antibiotic stewardship, total antibiotic consumption showed a significant upward trend during the study period. A consistent preference for cephalosporins, macrolides, beta-lactam-beta-lactamase inhibitor combinations, as well as parenteral preparations was observed.

## Introduction

How antibiotics are used is crucial for reducing the risks of antimicrobial resistance, which is one of the biggest threats to global health, food security, and development today [1]. Joint efforts have been made across the world to encourage prudent antibiotic use [2]. China is estimated to be the second largest consumer of antibiotics in the world [3], and given that it currently has the world’s largest population this gives China an important role in the process of constraining the use and misuse of antibiotics [4].

The Chinese government has made significant efforts to improve antibiotic use over the past decade [5]. These efforts were part of a series of measures undertaken in China, such as the introduction of national guidelines in 2004 [6], and the establishment of surveillance networks for both antibiotic use and antimicrobial resistance in 2005 [7]. In 2011, a three-year national level regulatory campaign was launched, which aimed to control total antibiotic use in secondary and tertiary hospitals [8]. This was legislated as a ministerial decree in 2012 [9]. In 2016, the National Health and Family Planning Commission, echoing the global governance endeavor of the WHO, announced a national action plan to combat antimicrobial resistance [10].

Antimicrobial stewardship in China has brought about a range of changes to antibiotic use. However, given the fragmentation of health care information data, aside from one study conducted in Shanghai [4], no reliable and comparable antibiotic use data at a population level nationwide have been published so far. Our study was aimed to investigate the trends and patterns in antibiotic use in China over a 5-year period.

## Materials and methods

### Study design and setting

We retrospectively analyzed aggregated monthly surveillance data on antibiotic sales to 468 hospitals from 28 provinces in China from January 2011 to December 2015. The data was derived from the electronic database of China Medical Economic Information (CMEI). CMEI is one of the largest government-approved information consultation and service platforms, which was designed to collect and analyze hospital medication usage under the administration of the China Pharmacy Association (CPA). The database covers more than 1,000 city-level public hospitals across mainland China. The sales of participating hospitals accounts for approximately 40% of total drug sales at city level public hospitals in China. Hospitals were sampled hierarchically based on geographical and socio-economic factors. We selected 468 tertiary hospitals in 28 provinces on the basis that they each had full records of antibiotic use during the five year study period which accounts for 72.6% of the overall sample hospitals listed in the CMEI.

### Data collection

Monthly antibiotic sales records data were extracted from the CMEI electronic database. Hospital information linked to the CMEI including the various types of generic antibiotics, amount of sales, the route of administration, expenditure, and geographical data were collected. Hospital names were concealed so as to protect confidentiality.

### Data management and analysis

Data were managed and analyzed in Microsoft Excel 2013. Sales data were extracted according to Anatomical Therapeutic and Chemical (ATC) classification J01 (i.e. antibacterial for systemic use) with defined daily dose (DDD) as measurement unit, as recommended by the WHO Collaborating Center for Drug Statistic Methodology [11]. A total of 173 unique chemical substance names were identified in single or combination antibiotics. These antibiotics were aggregated into 31 ATC-4 classes then into 9 ATC-3 groups.

To make the consumption data available to provide internationally comparable information, we adopted European Surveillance of Antimicrobial Consumption (ESAC) methods that is projected to collect data on antimicrobial consumption in ambulatory care and hospital settings in Europe [12]. The antibiotic consumption data were then converted into DDD per 1,000 inhabitants per day (DID) at the level of the active substance. Since there is no solution to directly achieve the exact number of population which our sample hospitals had covered, we adopted the following equation to calculate the weighted population as a proxy under two assumptions. First, there was no significant difference in the distribution of the sample hospitals across the provinces; second, there was no significant difference in the distribution of the population which was covered by the sample hospitals across the provinces. When exploring the distribution of antibiotic consumption in different regions in China, we adopted two different denominators: total population in a given year (Eq 1) and total inpatient volume in tertiary hospitals in a given year (Eq 2), to better reflect the patient flow between provinces. This way we could better describe the distribution of antibiotic consumption between different regions.

$$Y=P_{i}*\frac{n_{i}}{N_{i}}*\frac{{n_{i}}^{\prime }}{{N_{i}}^{\prime }}$$ (1)

$$Y^{\prime }=Q_{i}*\frac{n_{i}}{N_{i}}$$ (2)

*Y*: Coverage inhabitants in a given year in province i using census population data;
*Y*′: Coverage inhabitants in a given year in province i using inpatient volume;
*P*_*i*_: Total population in a given year in province i;
*Q*_*i*_: Total inpatient volume in total tertiary hospitals in a given year in province i;
*n*_*i*_: Number of sample hospitals in province i;
*N*_*i*_: Number of total tertiary hospitals in province i;
*n*_*i*_′: Number of total inpatients in tertiary hospitals in province i;
*N*_*i*_′: Number of total inpatients in all hospitals in province i;

All the relevant census data for calculating inhabitants were collected from China Health Statistics Yearbook and China Statistics Year Book [13].

### Comparison with ESAC data

Because of different health care systems and data availability, we were not able to distinguish the outpatient and inpatient use of antibiotics sales in the CMEI dataset. Thus, we compared the total antibiotic consumption in China with outpatient consumption in 29 European countries in 2012 when assessing the quality of trends and pattern of antibiotic consumption [14]. It was estimated that outpatient use accounted for 90–94% of the total antibiotic use for the countries ESAC dataset [15,16].

We adopted the 12 ESAC drug-specific quality indicators to assess the appropriateness of the patterns of total antibiotic use in China [17]. The number of antibiotics that accounted for 90% of drug utilization and the proportion of parenteral antibiotics within total consumption were also assessed.

## Results

### Total antibiotic use

**Fig 1:**
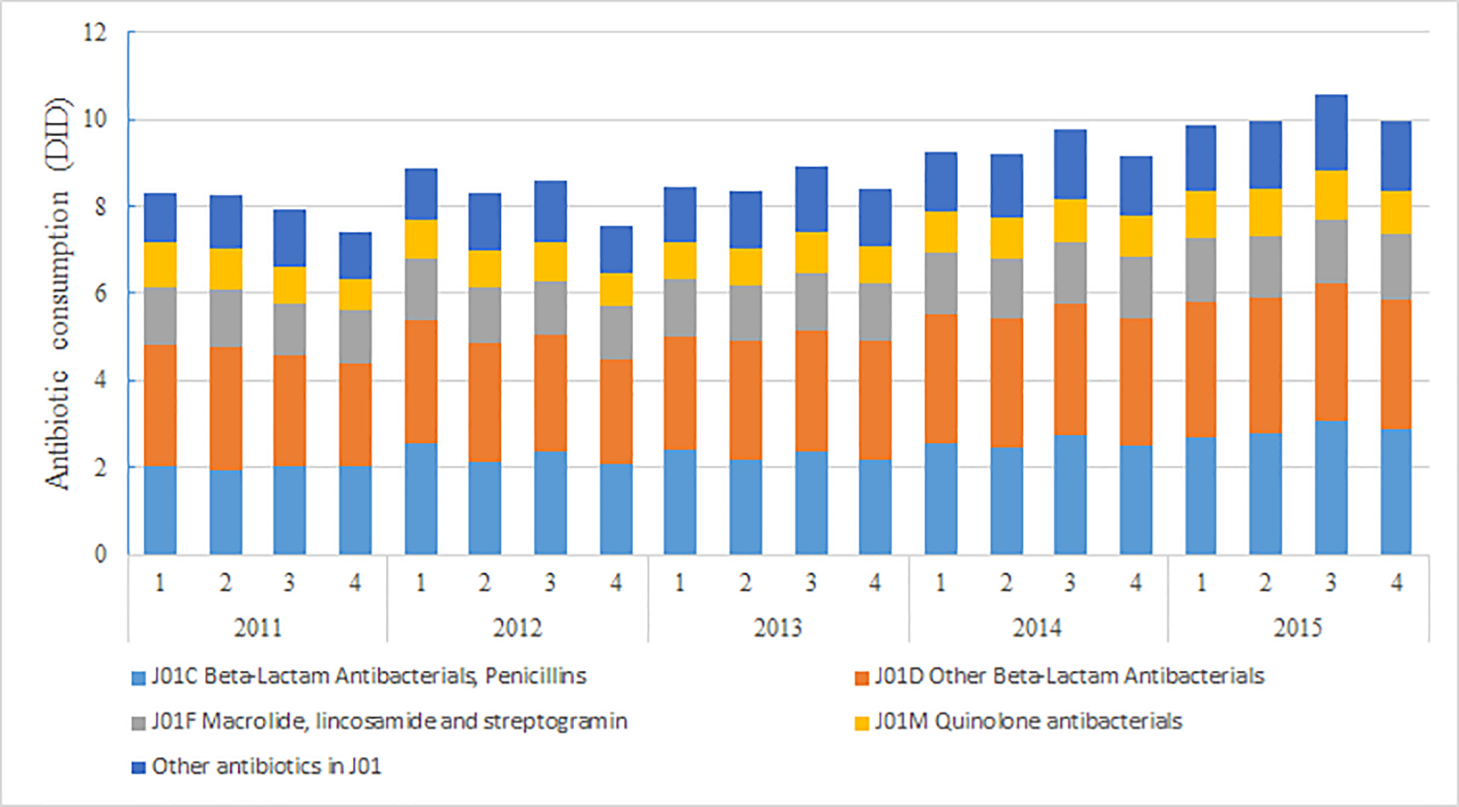
Consumption of antibiotic in China, 2011–2015.

Total antibiotic consumption in general, including all the specific antibiotic classes except for aminoglycoside antibacterials, significantly increased during the study period from an average of 7.97 DID in 2011 to 10.08 DID in 2015 (Fig 1 and S1 Table). The proportion of total pharmaceutical expenditure spent on antibiotics fell consistently from 21% in 2011 to 15% in 2015, while total pharmaceutical expenditure in China increased by an average of 5.5% in this 5-year period.

**Fig 2:**
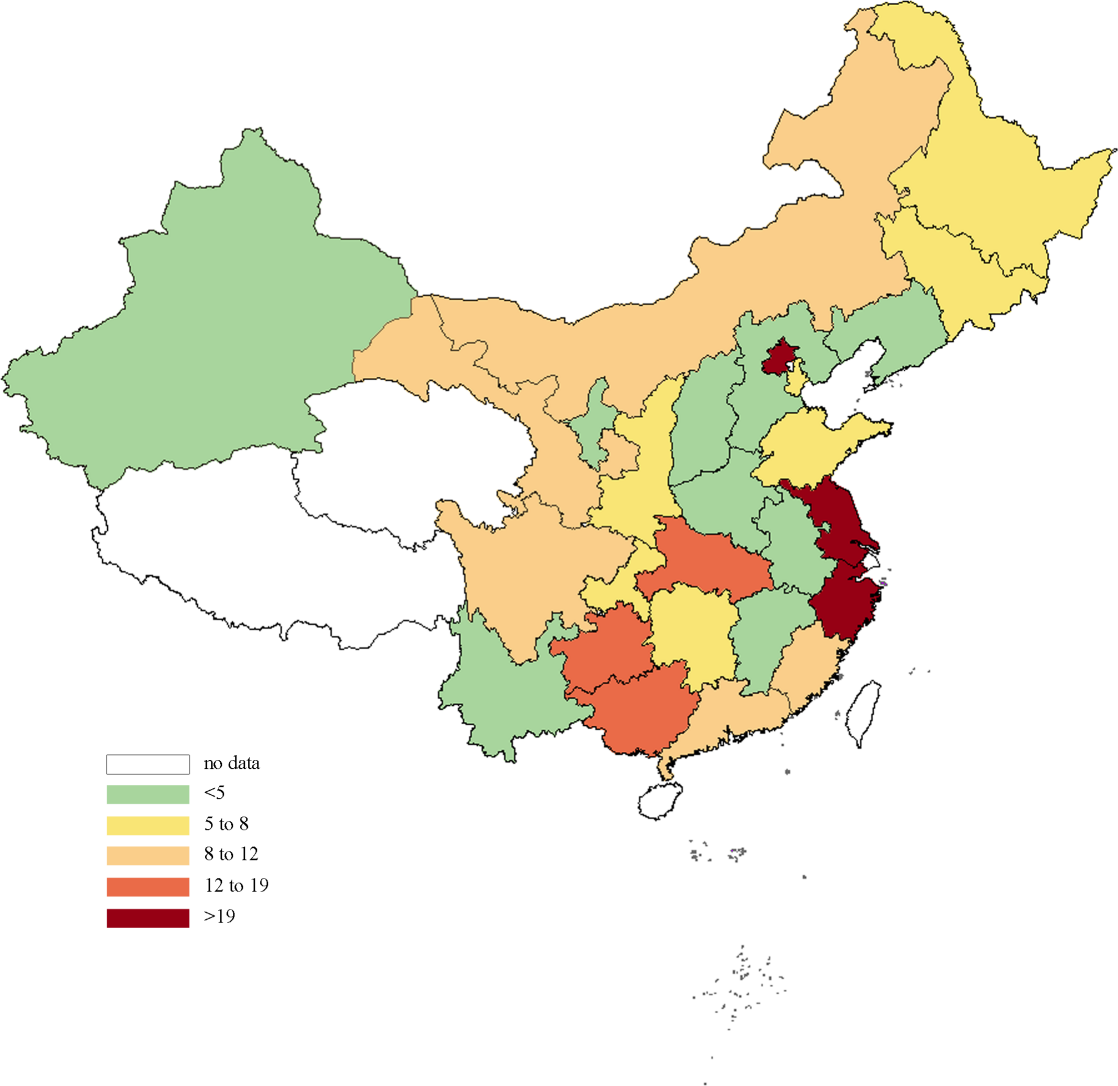
Consumption of antibiotics in China in 2015, expressed in DID.

The regional distribution of antibiotic consumption in China in 2015 was shown in Fig 2. Southern provinces used more antibiotics than the northern region, and the eastern coastal areas consumed the most antibiotics.

### Patterns of antibiotic use

The most frequently used antibiotic class in China were cephalosporins which accounted for 28.6% of total consumption, followed by beta-lactam-beta-lactamase inhibitor combinations with 20.0%, macrolides with 17.4%, and fluoroquinolones with 10.5% (all figures from 2015).

During the study period, cephalosporin consumption consistently increased from 2.46 DID to 2.88 DID, among which second and third generation cephalosprins contributed the most. The same trend was also observed in penicillin, macrolides, and carbapenems. The consumption of beta-lactam-beta-lactamase inhibitor combinations increased the most among all antibiotics classes by 0.66 DID. The consumption of aminoglycoside was the only antibiotic class that did not increase (Table 1).

**Table 1:** Antibiotic consumption by ATC-4 classifications in China during 2011–2015^a^.

	2011	2012	2013	2014	2015
**J01A TETRACYCLINES**					
**J01AA Tetracyclines**	0.20	0.21	0.22	0.26	0.29
**J01C BETA-LACTAM ANTIBACTERIALS, PENICILLINS**					
**J01CA Penicillins with extended spectrum**	0.32	0.33	0.34	0.38	0.39
**J01CE Beta-lactamase sensitive penicillins**	0.30	0.32	0.36	0.39	0.43
**J01CF Beta-lactamase resistant penicillins**	0.02	0.02	0.02	0.02	0.02
**J01CR Combinations of penicillins, incl. beta-lactamase inhibitors**	1.36	1.62	1.56	1.78	2.02
**J01D OTHER BETA-LACTAM ANTIBACTERIALS**					
**J01DB First-generation cephalosporins**	0.32	0.32	0.31	0.31	0.30
**J01DC Second-generation cephalosporins**	1.12	1.21	1.25	1.37	1.35
**J01DD Third-generation cephalosporins**	0.97	0.93	0.98	1.07	1.18
**J01DE Fourth-generation cephalosporins**	0.05	0.04	0.03	0.04	0.04
**J01DF Monobactams**	0.05	0.05	0.02	0.02	0.02
**J01DH Carbapenems**	0.06	0.08	0.09	0.11	0.15
**J01DI Other cephalosporins and penems**	0.01	0.00	0.01	0.01	0.01
**J01E SULFONAMIDES AND TRIMETHOPRIM**					
**J01EC Intermediate-acting sulfonamides**	0.02	0.02	0.02	0.02	0.03
**J01F MACROLIDES, LINCOSAMIDES AND STREPTOGRAMINS**					
**J01FA Macrolides**	1.19	1.23	1.24	1.35	1.41
**J01FF Lincosamides**	0.06	0.07	0.06	0.06	0.07
**J01G AMINOGLYCOSIDE ANTIBACTERIALS**					
**J01GA Streptomycins**	0.01	0.01	0.01	0.01	0.01
**J01GB Other aminoglycosides**	0.15	0.13	0.13	0.14	0.14
**J01M QUINOLONE ANTIBACTERIALS**					
**J01MA Fluoroquinolones**	0.88	0.83	0.87	0.96	1.06
**J01X OTHER ANTIBACTERIALS**					
**J01XA Glycopeptide antibacterials**	0.02	0.02	0.03	0.03	0.04
**J01XC Steroid antibacterials**	0.02	0.02	0.02	0.03	0.03
**J01XD Imidazole derivatives**	0.26	0.25	0.25	0.27	0.28
**J01XE Nitrofuran derivatives**	0.02	0.01	0.03	0.03	0.05
**J01XX Other antibacterials**	0.47	0.58	0.63	0.66	0.73

^a^Expressed in DDDs per 1000 inhabitants and per day.

Antibiotics categorized under J01BA, J01XB and J01RA are negligible and thus not listed herein.

**Fig 3:**
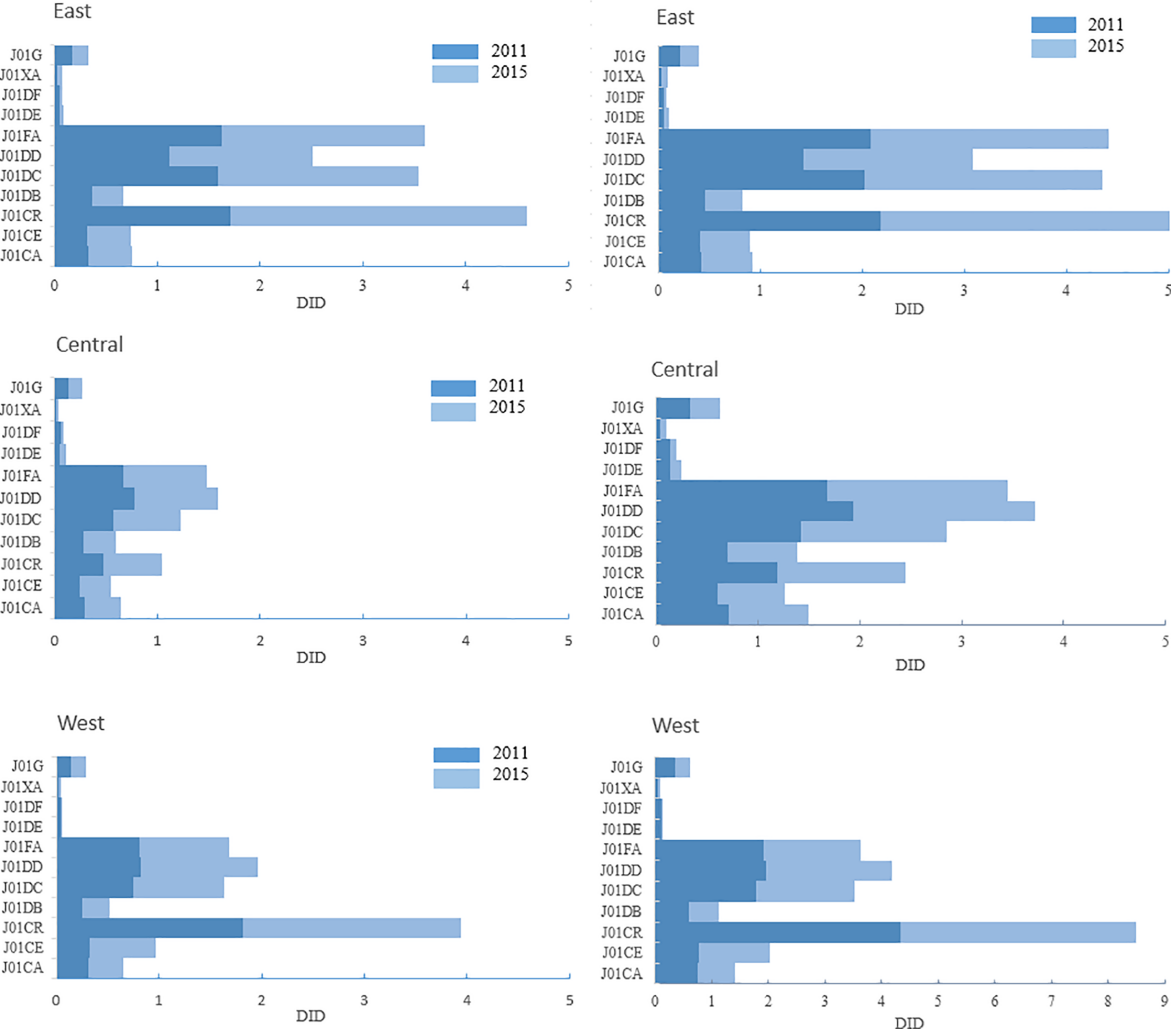
Commonly used antibiotic consumption in different regions of China by class in 2011 and 2015. Left: calculated based on population. Right: calculated based on inpatient volume. J01G: Aminoglycosides, J01XA: Glycopeptides, J01DF: Monobactams J01DE: Fourth-generation cephalosporins, J01FA: Macrolides, J01DD: Third-generation cephalosporins, J01DC: Second-generation cephalosporins, J01DB: First-generation cephalosporins, J01CR: Combinations of penicillins, incl. beta-lactamase inhibitors, J01CE: Beta-lactamase sensitive penicillins; J01CA: Penicillins with extended spectrum.

When exploring antibiotic consumption in different regions of China, we used two different denominators to better reflect the distribution in different regions, that is, population based and inpatient volume based (Fig 3). When using the population based denominator, it demonstrated that eastern regions consumed the most antibiotics, followed by the western regions and the central regions. However, the using inpatient volume based denominator showed a reverse result, namely the western regions consumed the most, followed by the eastern and central regions. Combinations of penicillins, including beta-lactamase inhibitors were the most consumed antibiotic class both in the eastern and western regions, while third-generation cephalosporins were used the most in the central region (Fig 3).

The number of antibiotics that accounted for 90% of total antibiotic consumption fell from 58 in 2011 to 43 in 2015. Half of these 43 antibiotics in 2015 were cephalosporins, among which 11 were only available in parenteral form (S2 Table).

**Fig 4:**
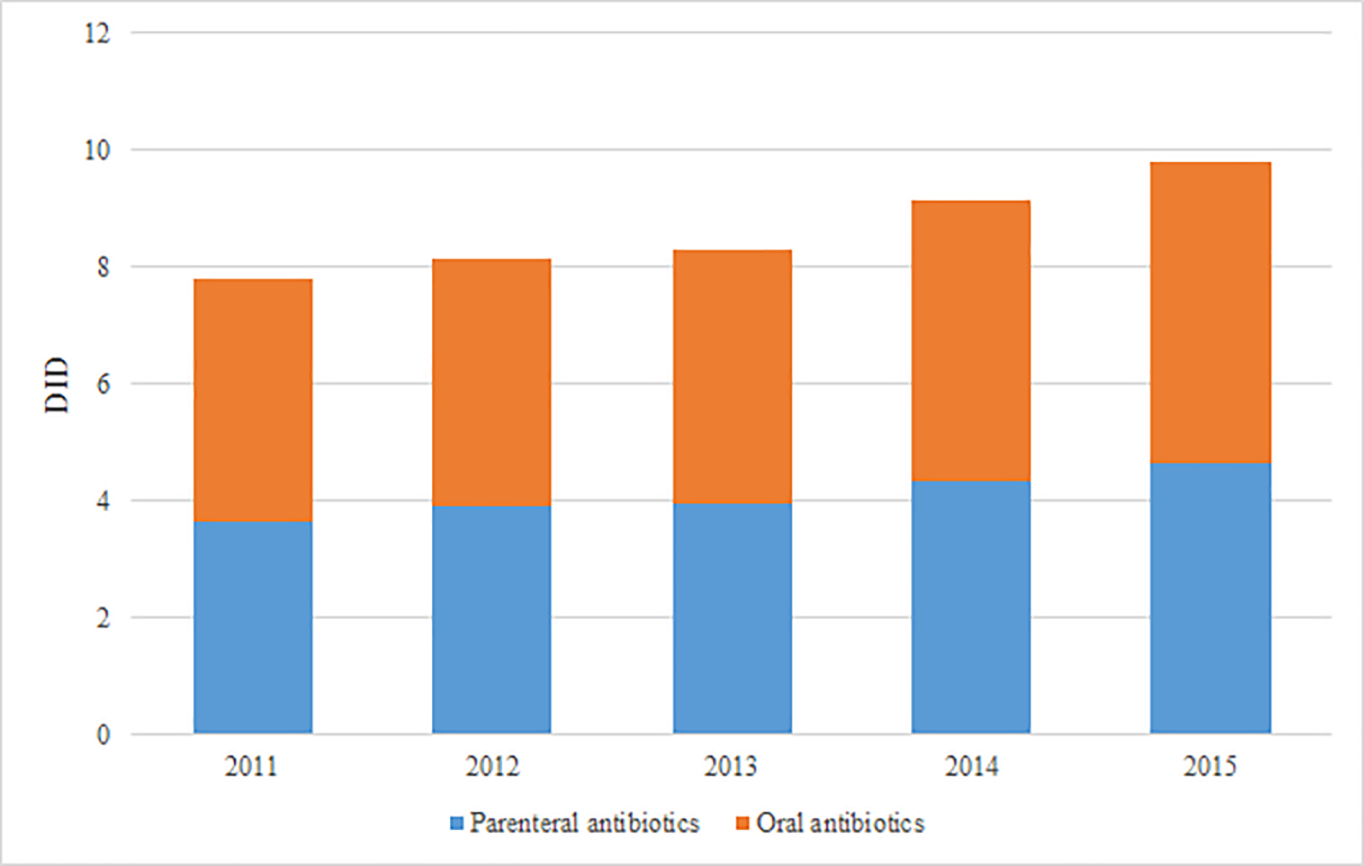
Consumption of oral and parenteral antibiotics in China, 2010–2015.

Total parenteral use of antibiotics in China in 2015 accounted for 47.1% of total DID, whereas the expenditure of parenteral antibiotics accounted for 87.0% of total drug expenditure. The proportion of antibiotics used in parenteral form was stable during study time period. The oral antibiotics consumption continuously increased from 4.13 DID in 2010 to 5.20 DID (Fig 4).

### Comparison of antibiotic consumption in China and Europe

We compared antibiotic use in China with ESAC data from 2012 (S3 Table). The comparisons showed that there was higher per capita consumption percentage in China during the study period than in at least 75% of the 29 European countries in terms of the consumption of third- and fourth-generation cephalosporins, as well as consumption of the fluoroquinolones.

## Discussion

This study is the first to estimate population-based antibiotic consumption at national level in China, using ATC/DDD methodology for international comparison. Our study quantified antibiotic consumption changes over a five year period, giving us the opportunity to benchmark the gaps between antibiotic utilization in China and European countries, which would be informing for healthcare providers, decision-makers, as well as the public.

The consumption of antibiotics is a major driver for the development of antimicrobial resistance [18]. As Fig 1 demonstrated, antibiotic consumption in China presented an increasing trend during the study period. This could be partly explained from the perspective of the pharmaceutical market and inpatient volume. Firstly, although the overall growth rate is decreasing, China’s antibiotic market sales at city level public hospitals continuously increased from 2011 to 2015 [19]. Secondly, the inpatient volume at China’s tertiary hospital almost doubled during the study period according to China Health Statistics Yearbook [20]. Despite the implementation of antibiotic stewardship in 2011, a combined with a consecutive three years national campaign [21], achieved a significant progress during the past few years [4,22,23]. For instance, antibiotic prescription rates in 65 public general hospitals reduced from 62.9% to 12.9% in inpatient and outpatient settings during 2010–2014 and total antibiotic use across all healthcare settings in Shanghai reduced by 25% in 2010 to 17.8 DID in 2012 (31% reduction), China still is a global leader of antibiotic usage [3,24]. When compared with the EU/EEA, we found that population-weighted antibiotic consumption in China’s tertiary hospitals was lower than EU/EEA mean consumption from the community of 22.4 DID, but much higher than the EU/EEA mean consumption from the hospital setting of 2.1 DID [25]. This may indicate that there was still a gap in antibiotic usage in the hospital setting between China and Europe, and more inappropriate antibiotic use might take place in the primary healthcare setting in China.

Cephalosporin was the most consumed antibiotic class of total antibiotic consumption which accounted for nearly 30% in China in 2015. This was observed in previous study conducted in other regions of China [26], as well as national surveillance data [27]. This was different with studies conducted in Europe [28–30] and United States [30], with more penicillin been prescribed than cephalosporin. Other than different diseases spectrum, one possible reason for this disparity might be that majority of the antibiotics were used in inpatient setting in China rather than outpatient setting [4], whereas ambulatory care setting accounted for most of the antibiotic consumption in high-income countries [15,20,31,32]. Another possible explanation is that cephalosporin has been recommend by national guidance for majority of the perioperative prophylaxis in China [33]. We found that quinolones were used more than penicillins in China. This might be because that skin testing is an obligatory requirement for penicillin prior to administration for allergy assessment in China [34], which may lead physicians more preferable to prescribe quinolones and cephalosporins in order to avoid this time-consuming test.

Carbapenems were considered as last-line treatment for multi-resistant bacteria. Although the proportion of consumption of carbapenems out of total antibiotic consumption is lower than EU/EEA data (1.5% verses 2.9%) [14], we still cannot ignore the fact that carbapenem consumption significantly increased more than a fold during the study period from 0.06 DID in 2011 to 0.15 in 2015. Since carbapenems is highly restricted and requires pre-authorization before use in China, the increase might be in response to the rising prevalence of ESBL-producing pathogenic bacteria, which was identified in epidemiological surveillance studies [33].

Moreover, the distribution of antibiotic consumption in China revealed that the eastern regions consumed more antibiotics than the middle and western regions when using census population as denominator. This could be explained by the patient flow between provinces caused by unbalanced healthcare resources allocation. Since the eastern regions are more developed than the central and western regions, the healthcare resources, especially the best ones, are located more in cities like Beijing, Shanghai, and Guangzhou, which brought patients from other regions travelled to these more developed areas for treatment. In addition, this phenomenon may be greater for central regions rather than western regions due to the geographical distance between central and eastern regions is closer. Hence, when we used inpatient volume as denominator to verify the speculation, the result showed that western regions consumed the most antibiotics. Antibiotic consumption in eastern regions decreased while antibiotic consumption in central and western region increased compared with population based consumption. This would further confirmed that there was a large volume of patient flow for healthcare between provinces in China.

The number of antibiotics that accounted for 90% of total antibiotic consumption dropped from 58 in 2011 to 43 in 2015. This could be partly because that antibiotics were mandatorily classified into three categories based on the prescription rights of the physicians as part of the stewardship campaign in 2012. This measure made antibiotics harder to be prescribed. Parenteral antibiotics accounted for 40% of the total consumption, but 80% of total expenditure. This proportion was significantly higher than that of Europe of 7% [35]. Since injectable antibiotics were more expensive than the oral forms, this might contribute to a distortion in the selection of administration route in China [4].

This study has several important limitations. First, the hospital participation functions on a voluntarily basis instead of mandatory participation and therefore might not be representative. Second, primary care setting was not included which may cause bias on the pattern of antibiotic consumption since a number of parenteral antimicrobial agents was used in primary care setting. Third, the population denominator used in the study may underestimate antibiotic consumption as it cannot include cross-provincial patient flow as discussed. Finally, as the study analyzed sales data rather than clinical usage, we were unable to determine the appropriateness of antibiotic use at the individual level.

## Conclusion

This study used aggregated sales records data to analyze the antibiotic consumption in China’s tertiary hospitals over a five year period. Although significant efforts have been made through antibiotic stewardship in the past few years, total antibiotic consumption showed a significant upward trend during the study period. The patterns of antibiotic consumption demonstrated that there was a consistent preference for cephalosporins, macrolides, beta-lactam-beta-lactamase inhibitor combinations, as well as parenteral preparations. More efforts are needed in the future to investigate the quality of antibiotic prescriptions, especially in primary health care settings in order to reduce risks of antimicrobial resistance.

## Supporting information

S1 Table Trends analysis of antibiotic consumption in China during 2011–2015. (DOCX)

S2 Table Drug utilization 90% profile of antibiotic use in China, 2015. (DOCX)

S3 Table Quality indicators for antibiotic consumption in China during 2011–2015. (DOCX)

S4 Table Specific antibiotics included in the study under each ATC class. (XLSX)
