# Supplementary material for: Trends and patterns of antibiotic consumption in China’s tertiary hospitals: Based on a 5 year surveillance with sales records, 2011-2015
Source: PLoS One. 2017 Dec 27;12(12):e0190314. doi: 10.1371/journal.pone.0190314 (PMC5744988; doi:10.1371/journal.pone.0190314)
Supplement: S1 Table — (DOCX) [file pone.0190314.s001.docx]

## S1 Table. Trends analysis of antibiotic consumption in China during 2011-2015

| Antibiotic Class | Coefficient | |
| --- | --- | --- |
|  | *r* | *p* |
| J01A Tetracyclines | 0.52 | < 0.001 |
| J01B Amphenicols | -0.63 | < 0.001 |
| J01C β-Lactam antibacterials, penicillins | 0.74 | < 0.001 |
| J01D Cephalosporins | 0.46 | < 0.001 |
| J01E Sulfonamides and trimethoprim | 0.47 | < 0.001 |
| J01F Macrolides | 0.64 | < 0.001 |
| J01G Aminoglycoside antibacterials | -0.01 | 0.40 |
| J01M Quinolones | 0.28 | 0.009 |
| J01X Other antibacterials | 0.72 | < 0.001 |
| Total antibiotic consumption | 0.70 | < 0.001 |
