# Supplementary material for: Trends and patterns of antibiotic consumption in China’s tertiary hospitals: Based on a 5 year surveillance with sales records, 2011-2015
Source: PLoS One. 2017 Dec 27;12(12):e0190314. doi: 10.1371/journal.pone.0190314 (PMC5744988; doi:10.1371/journal.pone.0190314)
Supplement: S2 Table — (DOCX) [file pone.0190314.s002.docx]

S2 Table. Drug utilization 90% profile of antibiotic use in China, 2015

| ATC Classification | Ranking, active substance | DID | % | Parenteral (%) |
| --- | --- | --- | --- | --- |
| J01A Tetracyclines |  |  |  |  |
| J01AA Tetracyclines | 15, doxycycline | 0.20 | 2.16 | 28.87 |
|  | 27, minocycline | 0.06 | 0.66 | 100.00 |
| J01C β-Lactam antibacterials, penicillins |  |  |  |  |
| J01CA Penicillins with extended spectrum | 7, amoxicillin | 0.35 | 3.79 | 0.06 |
|  | 8, amoxicillin and clavulanate potassium | 0.35 | 3.76 | 8.60 |
|  | 43, mezlocillin/sulbactam | 0.04 | 0.43 | 100.00 |
| J01CE β-Lactamase sensitive penicillins | 5, benzathine benzylpenicillin | 0.39 | 4.24 | 100.00 |
| J01CR Combinations of penicillins, incl. β-lactamase inhibitors | 19, piperacillin/sulbactam | 0.11 | 1.17 | 100.00 |
|  | 1, piperacillin/tazobactam | 1.45 | 15.74 | 100 |
| J01D Other β-Lactam antibacterials |  |  |  |  |
| J01DB First-generation cephalosporins | 40, cefradine | 0.05 | 0.50 | 100.00 |
|  | 21, cefathiamidine | 0.09 | 1.01 | 100.00 |
|  | 35, ceftezole | 0.06 | 0.61 | 100.00 |
|  | 22, cefazolin | 0.07 | 0.77 | 100.00 |
| J01DC Second-generation cephalosporins | 16, cefprozil | 0.19 | 2.05 | 4.50 |
|  | 18, cefuroxime sodium | 0.13 | 1.39 | 100.00 |
|  | 6, cefuroxime axetil | 0.38 | 4.13 | 0 |
|  | 11, cefaclor | 0.23 | 2.48 | 0 |
|  | 17, cefmetazole | 0.15 | 1.61 | 0 |
|  | 29, cefminox | 0.06 | 0.63 | 100.00 |
|  | 23, cefotiam | 0.07 | 0.71 | 0 |
|  | 28, cefoxitin | 0.06 | 0.64 | 100.00 |
| J01DD third-generation cephalosporins | 41, cefpodoxime | 0.04 | 0.49 | 100.00 |
|  | 10, cefdinir | 0.26 | 2.83 | 0 |
|  | 32, cefodizime | 0.06 | 0.62 | 0 |
|  | 30, cefmenoxime | 0.06 | 0.63 | 100.00 |
|  | 9, cefixime | 0.29 | 3.09 | 0 |
|  | 20, cefoperazone/sulbactam | 0.10 | 1.12 | 100.00 |
|  | 25,cefoperazone/tazobactam | 0.06 | 0.68 | 0.04 |
|  | 34, ceftriaxone | 0.06 | 0.61 | 100.00 |
|  | 24, ceftazidime | 0.06 | 0.68 | 94.52 |
|  | 38, ceftizoxime | 0.05 | 0.54 | 92.93 |
| J01DH carbapenems | 36, meropenem | 0.06 | 0.60 | 100.00 |
|  | 31, imipenem/cilastatin | 0.06 | 0.63 | 100.00 |
| J01F Macrolides, lincosamides and streptogramins |  |  |  |  |
| J01FA macrolides | 4, azithromycin | 0.42 | 4.58 | 12.93 |
|  | 39, dirithromycin | 0.05 | 0.52 | 100.00 |
|  | 37, erythromycin | 0.05 | 0.59 | 100.00 |
|  | 3, clarithromycin | 0.62 | 6.69 | 0 |
|  | 13, roxithromycin | 0.21 | 2.28 | 0 |
| J01FF lincosamides | 42, clindamycin palmitate | 0.04 | 0.47 | 8.44 |
| J01G Aminoglycoside antibacterials |  |  |  |  |
| J01GB other aminoglycosides | 33, gentamicin | 0.06 | 0.62 | 100.00 |
|  | 26, etimicin | 0.06 | 0.67 | 100.00 |
| J01M Quinolone antibacterials |  |  |  |  |
| J01MA fluoroquinolones | 14, moxifloxacin | 0.20 | 2.19 | 100.00 |
|  | 2, levofloxacin | 0.76 | 8.20 | 33.82 |
| J01X Other antibacterials |  |  |  |  |
| J01XD imidazole derivatives | 12, ornidazole | 0.23 | 2.47 | 43.21 |
