# Supplementary material for: Trends and patterns of antibiotic consumption in China’s tertiary hospitals: Based on a 5 year surveillance with sales records, 2011-2015
Source: PLoS One. 2017 Dec 27;12(12):e0190314. doi: 10.1371/journal.pone.0190314 (PMC5744988; doi:10.1371/journal.pone.0190314)
Supplement: S3 Table — (DOCX) [file pone.0190314.s003.docx]

## S3 Table. Quality indicators for antibiotic consumption in China during 2011-2015.

| **Year** | **Consumption** | | | | | **Relative consumption** | | | | **Broad/**  **Narrow** | **Seasonal variations** | |
| --- | --- | --- | --- | --- | --- | --- | --- | --- | --- | --- | --- | --- |
|  | **J01_DID** | **J01C_DID** | **J01D_DID** | **J01F_DID** | **J01M_DID** | **J01CE_%** | **J01CR_%** | **J01DD+DE_%** | **J01MA_%** | **J01_**  **B/N** | **J01_**  **SV** | **J01M_SV** |
| **2011** | 7.97 | 2.01 | 2.63 | 1.25 | 0.88 | 3.77 | 17.09 | 12.81 | 11.10 | 0.90 | 0.51 | -6.92 |
| **2012** | 8.34 | 2.28 | 2.65 | 1.30 | 0.83 | 3.79 | 19.41 | 11.64 | 10.00 | 0.90 | -5.43 | -8.65 |
| **2013** | 8.51 | 2.28 | 2.71 | 1.31 | 0.87 | 4.23 | 18.33 | 11.89 | 10.21 | 0.78 | -2.62 | -2.70 |
| **2014** | 9.35 | 2.56 | 2.96 | 1.41 | 0.96 | 4.16 | 18.99 | 11.88 | 10.25 | 0.78 | -3.50 | -8.61 |
| **2015** | 10.08 | 2.86 | 3.08 | 1.48 | 1.06 | 4.23 | 20.03 | 12.07 | 10.51 | 0.78 | N/A | N/A |

**Total antibiotic sales data, including those to hospitals and primary healthcare facilities.**

With reference to the quartile distribution of outpatient antibiotic use in 29 European countries in 2012.

|  | **J01_DID** | **J01C_DID** | **J01D_DID** | **J01F_DID** | **J01M_DID** | **J01CE_%** | **J01CR_%** | **J01DD+DE_%** | **J01MA_%** | **J01_B/N** | **J01_**  **SV** | **J01M_**  **SV** |
| --- | --- | --- | --- | --- | --- | --- | --- | --- | --- | --- | --- | --- |
| **P0** | 11.34 | 4.42 | 0.03 | 0.63 | 0.42 | 0.0 | 0.02 | 0.0 | 2.08 | 0.17 | 11.67 | 0.41 |
| **P25** | 15.20 | 6.87 | 0.75 | 1.98 | 0.92 | 0.3 | 13.12 | 0.1 | 5.91 | 5.06 | 17.33 | 8.40 |
| **P50** | 19.91 | 9.22 | 1.56 | 2.94 | 1.37 | 2.8 | 22.76 | 0.4 | 7.44 | 9.46 | 29.37 | 10.93 |
| **P75** | 22.93 | 12.63 | 2.72 | 3.48 | 2.39 | 6.5 | 31.66 | 2.4 | 9.96 | 36.41 | 32.32 | 22.50 |
| **P100** | 31.92 | 17.41 | 6.45 | 7.67 | 3.55 | 27.9 | 41.12 | 6.8 | 13.75 | 258.32 | 55.01 | 32.92 |

Boxes were labeled using four colors, red in P75-p100, yellow in P50-p75, light green in p25-p50, and dark green in p0-p25.

The indicators mean the lower the better, except for J01CE_%.

**J01_DID:** consumption of antibacterials for systemic use (J01) expressed in DID;

**J01C_DID:** consumption of penicillins (J01C) expressed in DID;

**J01D_DID:** consumption of cephalosporins (J01D) expressed in DID;

**J01F_DID:** consumption of macrolides, lincosamides and streptogramins (J01F) expressed in DID;

**J01M_DID:** consumption of quinolones (J01M) expressed in DID;

**J01CE_%:** consumption of β-lactamase-sensitive penicillins (J01CE) expressed as a percentage;

**J01CR_%:** consumption of combinations of penicillins, including β-lactamase inhibitors (J01CR) expressed as a percentage;

**J01DD+DE_%:** consumption of third- and fourth-generation cephalosporins [J01(DD+DE)] expressed as a percentage;

**J01MA_%:** consumption of fluoroquinolones (J01MA) expressed as percentage;

**J01_B/N:** ratio of the consumption of broad-{J01[CR+DC+DD+(F-FA01)]}to the consumption of narrow-spectrum penicillins, cephalosporins and macrolides [J01(CE+DB+FA01);

**J01_SV:** Seasonal variation of the total antibiotic consumption (J01) of a 12-month period starting in July and ending the following June, expressed as percentage: [(DDD (winter quarters)/DDD (summer quarters)-1] x 100;

**J01M_SV:** Seasonal variation of quinolone consumption (J01M) of a 12-month period starting in July and ending the following June, expressed as percentage: [(DDD (winter quarters)/DDD (summer quarters)-1] x 100.
